# Filling the Gap: Neural Stem Cells as A Promising Therapy for Spinal Cord Injury

**DOI:** 10.3390/ph12020065

**Published:** 2019-04-29

**Authors:** Inês M. Pereira, Ana Marote, António J. Salgado, Nuno A. Silva

**Affiliations:** 1 Life and Health Sciences Research Institute (ICVS), School of Medicine, University of Minho, Campus de Gualtar, 4710-057 Braga, Portugal; inesmiguelmarquespereira@gmail.com (I.M.P.); anammarote@gmail.com (A.M.); asalgado@med.uminho.pt (A.J.S.); 2 ICVS/3B’s—PT Government Associate Laboratory, Braga/Guimarães, Portugal

**Keywords:** spinal cord injury, cell-based therapies, induced pluripotent stem cells, neural stem cells, clinical trials

## Abstract

Spinal cord injury (SCI) can lead to severe motor, sensory and social impairments having a huge impact on patients’ lives. The complex and time-dependent SCI pathophysiology has been hampering the development of novel and effective therapies. Current treatment options include surgical interventions, to stabilize and decompress the spinal cord, and rehabilitative care, without providing a cure for these patients. Novel therapies have been developed targeting different stages during trauma. Among them, cell-based therapies hold great potential for tissue regeneration after injury. Neural stem cells (NSCs), which are multipotent cells with inherent differentiation capabilities committed to the neuronal lineage, are especially relevant to promote and reestablish the damaged neuronal spinal tracts. Several studies demonstrate the regenerative effects of NSCs in SCI after transplantation by providing neurotrophic support and restoring synaptic connectivity. Therefore, human clinical trials have already been launched to assess safety in SCI patients. Here, we review NSC-based experimental studies in a SCI context and how are they currently being translated into human clinical trials.

## 1. An Overview on Spinal Cord Injury 

Spinal cord injury (SCI) is one of the most disabling and devastating neurological injuries. It is recognized as a global health issue priority due to its impact on patient quality of life, complexity, and expensive medical care (Global Burden of Disease Study 2016 [1]). In 2016, the number of new SCI cases was 0.93 million (0.78–1.16 million) with a prevalence of 27.04 million (24.98–30.15 million) cases. Although the global incidence is similar between genders, men have higher incidence when compared with women at ages 20–40 years. Moreover, as the global population tends to grow and the health care systems to improve, it is expected an increase in the absolute number of people living with SCI [1]. Upon trauma, SCI patients can be stratified according to the spinal cord level affected, from higher cervical lesions that lead to partial or full tetraplegia (paralysis of the four limbs) to lower lesions that lead to paraplegia (paralysis of the lower limbs) [2]. Moreover, SCI etiologies can be subdivided into non-traumatic and traumatic injury. Non-traumatic SCI occurs when an acute or chronic disease (such as tumor, infection, or degenerative disc disease) causes the primary injury in the spinal cord, while traumatic SCI results from an external physical impact sufficient to damage the spinal cord (for instance, originated by a car crash, falls, violence or sports-related accident). Curiously, it was estimated that falls are the main cause, accounting for more than 50% of the total incidence [1].

### 1.1. From Acute to Chronic Phase

**Figure 1:**
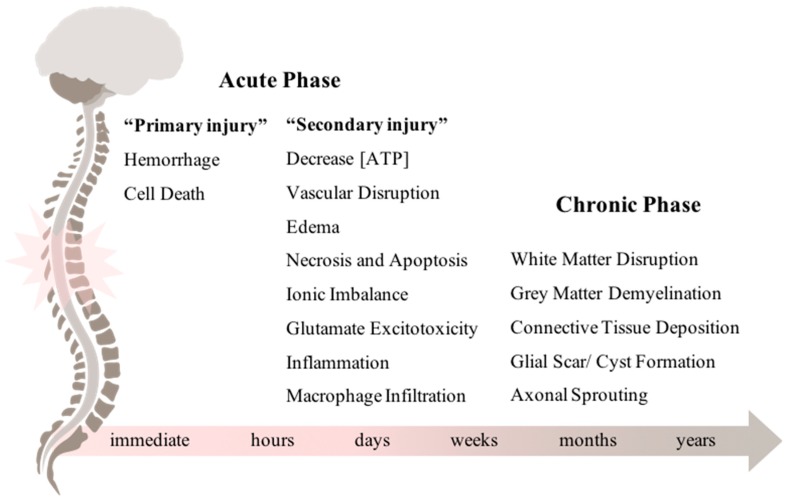
Underlying biological events during acute and chronic phases after spinal cord injury. The primary insult to the spinal cord immediately leads to a cascade of events that comprises the “secondary injury”. Weeks to months after the acute phase, the chronic phase is established and it may last throughout the patient’s life.

SCI can be described as a two-phase process: an initial acute phase divided in the “primary injury” and the “secondary injury”; and the chronic phase [3,4]. The “primary injury” depicts the time when the spinal cord is actually lesioned. Regarding the secondary events, it can occur over the time course of minutes to weeks, relying on a complex biological cascade of events that may aggravate the neurological outcomes. Finally, the onset of the chronic phase can occur days to months after the primary injury and continues throughout the patient’s life. Figure 1 represents the typical biological events of each phase.

The pathophysiology behind each phase has been extensively studied allowing us a better understanding of the biochemical and cellular events that occur after injury. The initial impact leads to immediate hemorrhage and cell death. Then, the “secondary injury” takes place, which was first suggested by Dr. Allen, in Philadelphia (1911). In his work, Dr. Allen reported improvements in the motor function of lesioned dogs after successfully removing the inflammatory fluid [5]. Thenceforth, extensive work has been developed to dissect the potential events that could be exacerbating the lesion severity during the acute phase [6]. These include vascular disruption, ischemia, edema [7,8,9], cell death (necrosis and apoptosis) [10], excitotoxicity, ionic imbalance [11,12], and a dysfunctional inflammatory response [13,14,15].

During the chronic phase there is dissolution of the grey matter, white matter demyelination, deposition of connective tissue, and the formation of the glial scar. In addition to all these physiological complications, many SCI patients also experience the development of pain syndromes [11] and mood disorders, like depression [16,17]. The glial scar is driven by reactive gliosis of astrocytes, microglia/macrophages, pericytes, and extracellular matrix molecules, acting as a physical barrier that limits axonal growth [18,19,20,21]. Myelin-associated proteins and proteoglycans are among the most inhibitory molecules in the central nervous system (CNS). Nogo-A, myelin-associated glycoprotein (MAG), and the chondroitin sulphate proteoglycans (CSPGs) have been the most studied ones [22,23,24]. Interestingly, several authors have already demonstrated that blocking such inhibitors in a SCI animal model leads to axonal regeneration and functional recovery [25,26,27].

Despite the inhibitory environment, after the injury, the spinal cord and the brain tend to reorganize and establish new circuits in a process called neuronal plasticity. Hollis and coworkers (2016) demonstrated that after a C5 dorsal column lesion in rats the motor cortex was able to remap when continuous training was performed. Moreover, when observing SCI patients, significant alterations in the brain reorganization were shown. In comparison with the control group, SCI patients showed a reduction in the sensorimotor cortex patterning activation under functional magnetic resonance imaging (fMRI) [28]. When looking to the spinal cord itself, several studies suggest that physical activity is one of the most critical influencers in neuronal plasticity. Regarding human studies, physical therapy is currently applied to incomplete SCI patients with positive results in patients’ recovery. 

Overall, SCI is a multi-dynamic process with no effective treatment mainly due to the failure of the CNS to successfully regenerate the damaged neuronal circuits. Therefore, researchers have been exploring novel therapies that can be translated into clinical meaningful recovery.

### 1.2. Clinical Management After SCI

Despite the major efforts currently made to develop new therapies for SCI, there is still no effective treatment. Numerous studies have reported some positive results in preclinical SCI models, however, translation to patients is still questionable and controversial. Typically, after trauma, the patient is completely immobilized and is constantly monitored to prevent possible complications, such as respiratory dysfunctions, cardiovascular aberrations, and hypoxia [29]. After stabilization, clinicians surgically decompress the spinal cord and control the lesion site [30]. 

The anti-inflammatory methylprednisolone sodium succinate (MPSS) was the first-line drug treatment for SCI patients [31]. Now spanning 50 years, a cordial discussion remains open regarding MPSS administration, safety, dosage, and time-administration [32,33]. Although neuroprotective effects were observed in preclinical studies as well as neurological improvements after its administration, the heightened risks of infection and death are a real concern for patients under MPSS treatment. In fact, the most recent guidelines from the Congress of Neurological Surgeons/American Association of Neurological Surgeons discouraged the administration of MPSS in acute SCI. Therefore, MPSS is not a viable long-term therapeutic choice, highlighting the need to develop new treatments targeting specific events that occur during the acute and/ or the chronic phase.

### 1.3. Novel Treatments for SCI

Pharmacologically, new neuroprotective alternatives have been targeting secondary events such as inflammation and excitotoxicity. Among them, minocycline and riluzole are two examples that are already under clinical trials. Minocycline, a semisynthetic tetracycline antibiotic, is classified as a neuroprotective agent by improving the exacerbated inflammatory microenvironment observed during secondary SCI [34]. After positive motor outcomes observed during phase I/II clinical trial, the pharmacological administration of minocycline was further encouraged to be tested in a phase III clinical trial (Minocycline in Acute Spinal Cord Injury (MASC)—NCT01828203) [35]. Riluzole, a benzothiazole anticonvulsant, showed to be able to block the abnormal glutamatergic transmission in neuronal synapses [36]. Preclinical studies strongly pointed out for a beneficial effect in neurological tissue preservation and motor recovery [37,38]. Consequently, and after the positive effects reported during the phase I/IIa clinical trial, riluzole is now involved in an international phase II/III multi-center clinical trial (Riluzole in Acute Spinal Cord Injury Study (RISCIS)—NCT01597518) [39,40].

Fibroblast growth factor-2 (FGF-2; or basic fibroblast growth factor) has been implied in different biological processes, such as limb and nervous system development, wound healing, and tumor growth, due to its function in cell proliferation and survival [41]. Regarding SCI, different reports have evidenced a role for FGF-2 in spinal cord neural stem and progenitor cells proliferation, angiogenesis, and glial cavitation reduction. While some SCI animal models shown hind limb improvements after intrathecal injection of FGF-2, other studies do not report any functional recovery benefits after treatment. Still, a human clinical trial is now being conducted to assess safety and efficacy of a specific biodegradable device with heparin-activated FGF1 in traumatic SCI patients (NCT02490501). The aim of the study is to develop a regenerative treatment option by taking advantage of the crucial role of FGF1 in neuroprotection and axon regeneration of the CNS [41,42]. 

Cytokines also exhibit interesting characteristics that can promote morphological and functional recovery of the spinal cord after trauma. The most widely studied cytokine for SCI treatment is the Glycoprotein Granulocyte Colony-Stimulating Factor (G-CSF), known to reduce inflammatory cytokine expression and to promote survival of ischemic cells [43]. Moreover, in a nonrandomized phase I/IIa clinical trial the administration of G-CSF demonstrated to be safe, leading to some improvements in the American Spinal Cord Injury Association (ASIA) scale [44,45]. Another promising cytokine for SCI treatment is interleukin-4 (IL-4). This cytokine is able to activate macrophages to a phenotype associated with repair [46]. Preclinical studies have demonstrated neuroprotection and motor recovery after the administration of IL-4 [47,48].

Besides these molecular approaches, stem cell-based therapies have shown great potential over the past decades, based on the rationale that transplanted cells can differentiate and substitute the lost tissue. Moreover, stem cell-based treatments can also modify the microenvironment and regenerate the damaged circuits. However, there are also some challenges inherent to cell transplantation, including: the choice of the cellular source, the establishment of differentiation protocols, and the monitoring of cell grafts survival and integration. 

Regarding stem cell transplantation, encouraging results have already been reported for spinal cord repair, being particularly relevant for neuronal and glial replacement, remyelination, connectivity restoration, stimulation of precursor cells, as a bridge of cysts/cavities, and for improving the expression of beneficial neurotrophins/cytokines. A wide plethora of different cell-types has already been tested and transplanted into the injured spinal cord, both in animals and humans, including Schwann cells [49], olfactory ensheathing glia [50], skin-derived precursors [51], mesenchymal stem cells (MSCs) [52], oligodendrocyte progenitor cells (OPCs) [53], and neural stem/progenitor cells (NS/PCs) [54]. Although all cell-types have intrinsic advantages but also limitations, the focus of the present review is directed for NSC-based therapies.

## 2. Stem Cells in SCI: Past, Present, and Future 

### 2.1. From the Embryo to A Structured Spinal Cord

**Figure 2:**
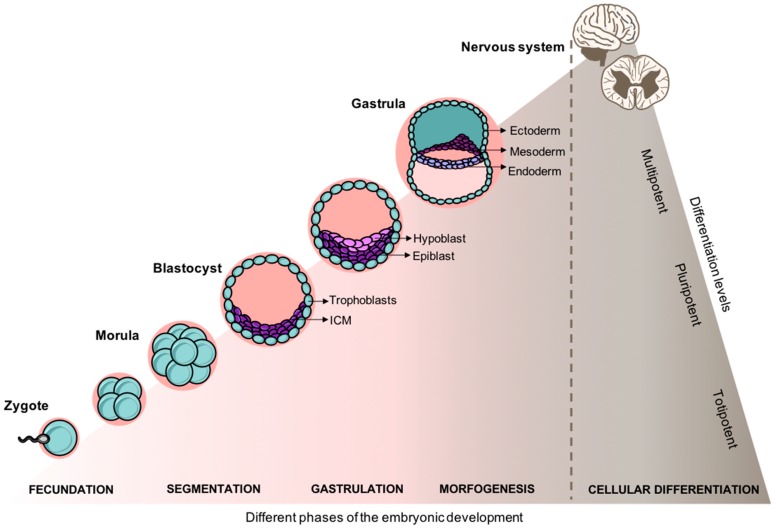
Embryonic development and cellular differentiation. After fecundation, the entire embryogenesis is spatially and temporally coordinated dynamically shifting the gene expression, cell growth, and cellular differentiation.

The human embryonic development of the nervous system is a complex and highly tuned-process. During the embryonic period, the primary three germ layers (ectoderm, mesoderm, and endoderm) are established as the basis of the various systems and organs of the body through numerous mitotic divisions. As illustrated in Figure 2, from a solid mass of totipotent cells—morula—the embryo develops into the blastocyst. The blastocyst is a pluripotent structure that consists of a cellular outer layer of trophoblasts and an inner Embryonic Stem Cell (ESC) population, the inner cell mass (ICM) [55]. From the ICM cells adjacent to the cavity, a new layer of flat cells is formed, the hypoblast, while the rest of them remain relatively undifferentiated and are termed as epiblast. Subsequently, the blastocyst becomes attached to the endometrium of the uterus, and epiblast cells migrate ventrally along the median plane to form the primitive streak. Embryonic cells begin to differentiate, replacing the hypoblast by the endoderm whereas the remaining part of the epiblast arises to the ectoderm [56]. This process is termed as gastrulation where the monolayered blastula forms a bilayer gastrula with the well-defined primary layers. It is from the ectoderm the notochordal process began, that which will ultimately will arise into the nervous system [57,58]. 

At approximately the 23rd day of development, the neural plate is already observed in the embryo initiating the neurulation process. As the neural plate begins to roll-up, it develops into the neural tube and consequently to the neural crest [58]. During this period, ESC-derived epiblast-like cells are redefined as neuromesodermal progenitors (NMPs) and are the cellular source for spinal cord development [59,60]. Timed and spatial expression pattern of *TBX6* and *SOX2* genes in NMPs drive cells into their mesoderm or neural fate [61]. Further, other specific patterning genes regulate the neural subtype fate of neural stem cells (NSCs) along the rostral-caudal and dorsoventral axis, in a concentration-dependent manner. While retinoic acid (RA) is highly involved in the activation of rostral homeobox (*HOX)* genes (*HOX1-5* paralog) responsible for a more broad brainstem-to-rostral cervical spinal cord identity, the balance between WNT and FGF signals induces a more caudal neuroaxis spinal HOX gene expression (*HOX6-9* paralog), specifically for a cervical and thoracic spinal cord identity [62,63,64]. Once the neurulation process is concluded, cells begin to differentiate into mature neurons, being the motor neurons the first ones to develop. Architectonic organization of the spinal cord becomes more and more complex and neurons, non-neurons, and fibers become myelinated for the development of the major tracts of the spinal cord. Fully maturated, the spinal cord is composed by the white matter (mostly myelinated axons) surrounding the gray matter (mostly interneurons, cell bodies, and glial cells). In the white matter the axons are organized in fiber tracts that run longitudinally through the spinal cord, ascending tracts transmit information from the periphery to the CNS and the descending tracts relay information from the brain to the rest of the body.

### 2.2. Historical Perspective of Cell-Based Research

**Figure 3:**
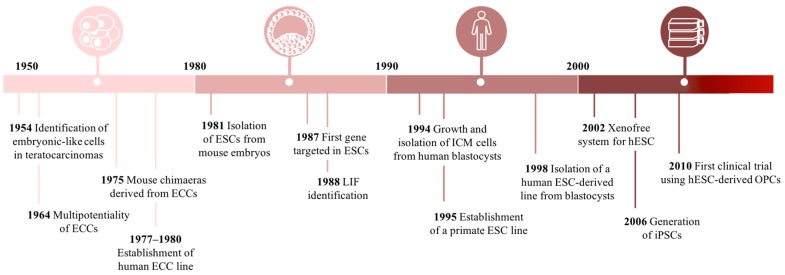
Timeline of embryonic stem cell (ESC)-based research. ICM: inner cell mass; OPC: oligodendrocyte progenitor cells; iPSCs: induced pluripotent stem cells; hESCs: human embryonic stem cells; ECCs: embryonal carcinoma cells.

Over the past decades, we have been witnessing to unprecedented and groundbreaking progress in cell-based research (Figure 3). The potential of such tools has been capturing the attention of the scientific community, clinicians, as well as the general public. The idea of innovative cell-based therapies to treat a wide spectrum of human diseases and traumas has been inspiring researchers. 

#### 2.2.1. Finding Embryonic Stem Cells

Cell-based research turning point begun in the 20th-century when Stevens and Little (1954) were deciphering the complexity of teratocarcinomas. These tumors contained a relatively undifferentiated cell-type known as “Embryonal Carcinoma Cells” (ECCs), long suspected as the stem cell of the tumor [65]. In the following decade, an emerging interest regarding ECCs was notorious, culminating in some important findings, namely: (1) a single tumor-derived cell is able to differentiate into all the heterogeneous cell types that are typically found in a teratocarcinoma [66]; (2) ECCs can be continuously expanded in vitro when co-cultured with inactivated mouse embryonic fibroblasts (MEFs); (3) after blastocyst ECC injection a chimeric mouse can be generated [67,68]; and (4) differentiation into any embryonic germ layer [69,70].

The ECCs therapeutic potential was compromised due to their tumorigenic potential and aneuploidy karyotype. In an attempt to overcome this drawback, in 1981 two independent laboratories reported the isolation and establishment of ESCs from early mouse embryos [71,72]. By resorting to pre-implanted blastocysts, Evans, Kaufman, and Martin surgically removed the ICM, a sharp source of pluripotent cells, and culture it on fresh feeder layers under conditioned medium. As a result, they obtained a normal diploid ESC line that could differentiate into all mature cell-types from the three germ layers in vitro, and in vivo [71,72]. In 1984, Andrews et al. and Thompson et al. resorted to Tera-2, the oldest extant cell line established from a human teratocarcinoma, to isolate and derive genetically identical clone cells. They observed that clones were highly adapted to culture overgrowth and could maintain their differentiation potential. Moreover, under retinoic acid exposure, clones were capable of differentiating into neuron-like cells and other somatic cell-types [71,72,73]. As the knowledge regarding pluripotency mechanisms improved, the derivation and differentiation protocols began to be more refined. For instance, Matsui et al. (1992) enhanced the long-term culture of ESCs by adding bFGF to the culture medium [74]. 

Considering the advances in animal derived-ESCs, the isolation and culture of human pluripotent cells became an exciting challenge at the time. In 1993, Bongso et al. described for the first time the development and maintenance of ICM cultures and the following isolation of ES-like cells from its center. Cells maintained a normal karyotype and stemness-like morphology, however, a limited number of clusters differentiated into fibroblasts [75]. In 1998, two groundbreaking works were reported the establishment of embryonic germ cell (EGC) and ESC lines isolation from a human source [76,77]. Shamblott et al. reported the isolation of PGCs from gonadal ridges and mesenteries at 5–9 weeks post-fertilization, which after a period of 7–21 culture days were positive for alkaline phosphatase activity and also for a commonly panel of immunological markers (SSEA-1, SSEA-3, SSEA-4, TRA-1–60, and TRA-1–81) used to characterize ESCs and EGCs [76]. Later, Thomson and his co-workers announced the derivation and establishment of ESCs in non-human primates. After several passages, the cells retained a normal karyotype, high levels of telomerase activity and typical pluripotency markers expression [77]. To obtain ESCs, researches were using blastocyst which is an embryonic structure that derives after fecundation which implies the use of human embryos. As expected, this idea started to provoke some discussion in the media due to the ethical and religious concerns involved. Despite the controversy, in the following years, different groups developed several ESC lines improving in vitro culture approaches and differentiation protocols. Traditionally, MEF feeders were used to support ESCs growth, however, the relevance of ESCs for a therapeutic approach motivate the reassessment of these support systems, considering the high risk of pathogens cross-transference from the animal feeder cells. A novel xeno-free system was suggested by Richards et al., (2002) with all animal-based products discarded, empowering the potential application of ESCs into the clinic [78].

Therefore, the first clinical trial using human ESC-derived OPCs was approved in 2010 (NCT01217008) [79,80]. The cells were transplanted into patients with spinal cord injury (SCI) and no major complications were reported regarding toxicity, allodynia, or tumor formation [81]. A phase I/IIa dose-escalation study was then initiated in 2014 (NCT02302157), and considering the updates from the responsible biotech company, OPCs were successfully engrafted into the spinal cord of patients. Moreover, some motor improvements were observed, but the official data for the entire study is expected to be published in the first quarter of 2019. 

In conclusion, some convergent points stood out and common ESCs characteristics were established among the scientific community: (1) source of pluripotent cell population; (2) maintenance of a stable and diploid karyotype; (3) indefinite propagation in a primary embryonic state; (4) differentiation into any mature cell-type of the three embryonic germ layers; and (5) expression of specific nuclear and cytoplasmic markers of pluripotency [82]. All these characteristics put ESCs forward as good candidates for a wide range of applications, including as a platform for organogenesis studies through the generation of complex tissues such as, the patterned neural tube [83], cerebral organoids [84], and a mature spinal cord [85]. 

#### 2.2.2. Searching for Pluripotency in Adult Tissues

Despite the excitement created around the ESCs therapeutic potential some issues were hampering their translation to the clinics, mainly, ethical concerns, tumorigenic potential, and impossibility for autologous transplantation. Adult stem cells, such as mesenchymal stem cells, were a viable alternative, however, their differentiation potential is limited. Thus, further efforts were pursued to find a source of pluripotent stem cells (PSCs). 

Unexpectedly, in 1962 Gurdon et al. reported the full development of a complete *Xenopus organism* by transferring the nucleus from a somatic cell to an enucleated egg [86]. Although in a very primitive way, Gurdon et al. explored for the first time the concept of reprogramming somatic cells into pluripotent cells. These developments paved the fundamentals of somatic cell nuclear transfer (SCNT) technique which allows the production of genetically suitable cells and tissues. Particularly, it was through SCNT that the famous “Dolly the sheep” was produced, a live offspring capable to mature into adulthood [87]. Despite the notable progress in SCNT research, human experiments were still intolerable due to the ethical concerns [88].

**Figure 4:**
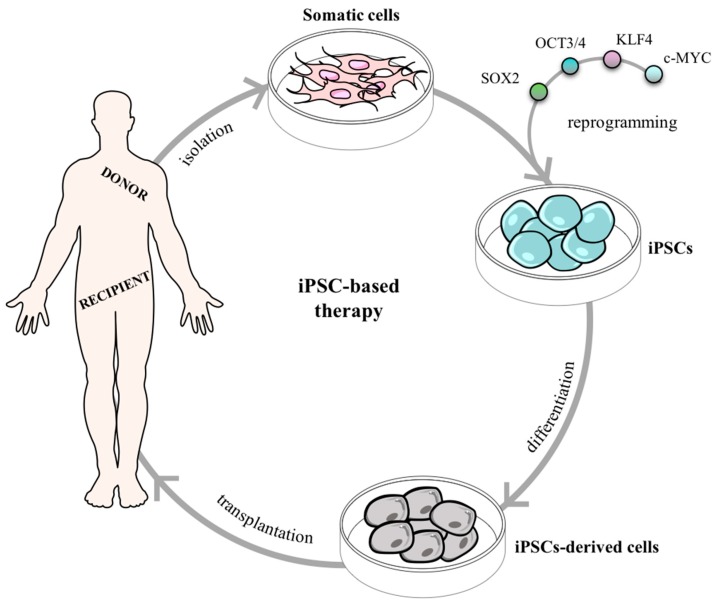
Somatic cells reprogramming using Takahashi and Yamanaka’s factors (SOX2, OCT3/4, KLF4, c-MYC) to induced pluripotent stem cells (iPSCs). Induced PSCs can further be used as a novel therapeutic strategy for cell transplantation.

Therefore, additional methodologies were explored to generate PSCs such as: parthenogenesis [89], cell fusion [90], direct somatic cell reprogramming [91], and testis-derivation [92], (see the Muller and Lengerke (2009) review [93]). Among all, when pros and cons are balanced direct somatic cell reprogramming shows to be the most promising technique opening up unprecedented opportunities for regenerative medicine. The groundbreaking report was originally published by Takahashi and Yamanaka in 2006 where they remarkably demonstrated the generation of induced PSCs (iPSCs) from mouse skin fibroblasts. After a full genomic screening, Takahashi and Yamanaka filtered 4 out of 24 genes as pivotal transcriptional factors for the induction and maintenance of iPSCs in an ES-like state: Oct3/4, Sox2, c-Myc, and Klf4 (Figure 4). As a proof of concept, the particular features of ESCs were also present in iPSCs, namely: (1) resembling morphology and growth properties; (2) expression of specific gene markers; (3) tumor formation after transplantation in nude mice; (4) tissue differentiation from all the primary germ layers in the tumor; and (5) complete embryonic development after mouse blastocysts injection [91]. Overall, Takahashi, and Yamanaka created important foundations in regenerative medicine by producing autologous cells and thus, avoiding the need for immunosuppressive agents after transplantation. This pioneering work was the base for the recognition in 2012 with the Nobel Prize in Physiology or Medicine award for Shinya Yamanaka. 

Despite all the benefits, there are some inherent limitations of iPSCs that must be kept in mind to ensure safety (Table 1), such as the potential risk of inducing genomic and/or epigenomic alterations during iPSCs reprogramming [94,95]. Nevertheless, over the past years, rapid and extensive research has been done to improve iPSCs technology to be applied in regenerative medicine, drug therapy, and disease modeling [96].

The generation of iPSCs have already been done from a wide range of tissues, from adult human fibroblasts [91,97] to liver and stomach [98,99]. In 2007, Yu et al. modified the gene set used by Takahashi and Yamanaka and instead of the tumorigenic c-MYC, the authors used Lin-28 obtaining a similar population of iPSCs [100]. Moreover, Nakagawa et al. (2008) observed that supplementation with c-Myc was not crucial for iPSCs induction and although the authors reported a significantly lower reprogramming efficiency, they reduced down to zero the probability of teratoma formation after transplantation [101]. 

On the contrary to ESCs, the use of iPSCs as a valuable source for cell transplantation was well accepted mainly due to the absence of ethical concerns. However, iPSCs also underwent some challenges; namely by the inappropriate use of animal-derived components and the use of retroviral vectors to transfect somatic cells. Thus, novel integration-free methods and xeno-free cultures were developed as well as innovative virus-free strategies [102,103]. These latter advances prompted exponentially the therapeutic applicability of iPSCs by decreasing the risk of tumorigenicity associated with viral genomic-integration.

In 2014, Dr. Takahashi and his team (RIKEN, Kobe, Japan) submitted the first-in-human trial using iPSCs (funded by Highway Program for Realization of Regenerative Medicine and others; University Hospital Medical Information Network Clinical Trials Registry [UMIN-CTR] no. UMIN000011929). A patient with neovascular age-related macular degeneration (AMD), a degenerative retinal disease, received a transplant of a sheet of retinal pigment epithelial (RPE). The RPE cells were differentiated using autologous iPSCs from skin fibroblasts and no immunosuppressive agents were administrated to the patient. After transplantation, neither signs of immune rejection were observed nor aberrant genomic alterations, suggesting no tumorigenesis of the cell graft [104]. These positive outcomes highlight all the potentialities behind iPSC technology as a novel tool for cellular therapy and have launched the beginning of more clinical trials targeting many other conditions (see the Kavyasudha et al., 2018 review [105]). 

Human iPSCs are mainly obtained from skin fibroblasts however, a skin biopsy may not be the best source due to the risk of infection and scar formation [97,106]. Thus, other less invasive somatic cell sources have been considered, such as peripheral blood due to the minimal procedures necessary to collect it. In fact, clinicians and researchers have strong expectations in peripheral blood as a gold standard clinical-grade source of iPSCs for the future [80,107]. 

Additionally, iPSCs technology has also been explored for disease modelling. The establishment of some disease-specific iPSCs lines have already allowed a better understanding of the mechanisms that trigger and are behind certain diseases [108]. The first successful report was based on the generation of iPSCs from a child with Spinal Muscular Atrophy (SMA) [109]. Studies on drug screening and validation have also been conducted using patient-specific cell lines, and relevant advances have already been achieved, for instance, in Rett syndrome [110], and in Fanconi anemia [111]. 

In conclusion, iPSCs represent a very flexible technology that opened new and challenging opportunities to develop cell-based therapies due to its ability to differentiated into any cell-type given the right culture conditions. The numerous advantages of induced stem cells, from no immune rejection to high reproducibility, prompted the field to employ them in patient-specific diseases studies, developmental studies, cell transplantation as well as in drug screening. As a consequence, we are now moving fast towards a more personalized medicine.

#### 2.2.3. Neural Stem Cells

In the past, researchers assumed that neurons were only generated during embryogenesis, however, Altman and Das (1964) published a remarkable report describing the generation of new neurons in the hippocampal dentate gyrus of an adult rat brain [112]. The generation of new neurons from NSCs is a highly tuned process during nervous system development. Nevertheless, neurogenesis also occurs throughout adult life as an extremely limited process that attempts to sustain a proper balance between self-renewal and differentiation [113,114]. In the adult brain, the two best described niches that harbor NSCs are the subventricular zone (SVZ) and the periventricular region of the spinal cord [113,115]. 

**Figure 5:**
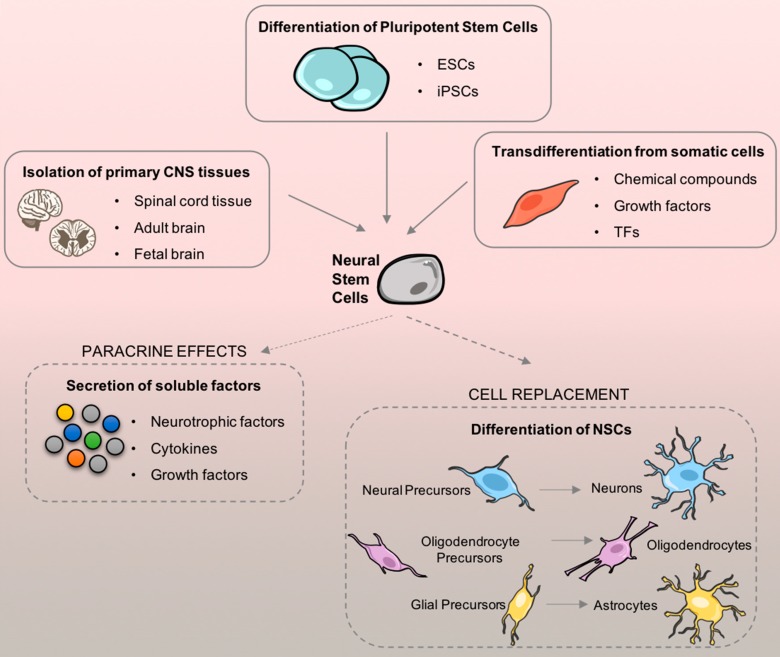
Neural stem cell (NSCs) sources and their therapeutic applicability after cell transplantation. There are three main sources to generate NSCs: isolation from primary central nervous system (CNS) tissue; differentiation of pluripotent stem cells, and lineage reprogramming of somatic cells. After obtaining a considerable number of cells that fulfill the needs of cell transplantation cells can be applied. Once on the injury site, NSCs can be differentiated and also secrete paracrine factors that may also support neurological repair.

Therefore, the idea of transplanting NSCs is a very promising therapeutic approach for many neurodevelopmental, neurodegenerative, and neurotraumatic diseases by replacing and reestablishing damaged neuronal circuities [114]. Thus, one of the first topics that should be discussed is how would NSCs be obtained taking in consideration both the cell source and the cell dosage required for adequate transplantation. Until this point, there are three main sources: direct isolation from primary CNS tissue (either from the fetal or adult brain); differentiation from pluripotent stem cells; and, transdifferentiation from somatic cells (Figure 5). 

***NSCs from CNS primary tissues.*** In 1992, Reynolds and Weiss were the first to report the isolation and culture of NSCs in spherical clusters (neurospheres) from the striatal neurogenic area of an adult mouse brain [116]. Two years later, they remarkably demonstrated in vivo that the subependymal region is a viable source of NSCs in the mouse brain [117]. Concomitantly with their work, two specific mitogenic growth factors, the epidermal growth factor (EGF) and the bFGF were identified as critical factors that cooperatively induce self-renewal, proliferation, and expansion of NSCs in clonal aggregates [115,116]. Indeed, after isolation, NSCs can grow into single-cell suspensions that ultimately form regular neurospheres. These non-adherent neurospheres are particularly interesting due to their self-renewal capacity and because of their capability of establishing a favorable extracellular-matrix microenvironment that helps to maintain stemness. Moreover, neurospheres can be sub-cultured and expanded to increase the available pool of cells. Adult NSCs have, however, the disadvantage of not being able to be used as an autologous cell source.

***Differentiation of NSCs from pluripotent stem cells.*** PSCs have been used as a very attractive alternative to primary tissue isolation. Differentiation of NSCs from ESCs and iPSCs gained a lot of attention due to their therapeutic applicability potential. As previously discussed, iPSC-derived NSCs have striking advantages over ESC-derived NSCs, namely the possibility of autologous transplantation. Regarding differentiation protocols, PSCs have been cultured either as embryoid bodies (EB) or in monolayer cultures. Considering EBs, they grow in suspension and can be differentiated into neural tube-like rosettes and, subsequently, into NPCs under specific conditions [118]. More recently, differentiation protocols of NSCs using iPSC-derived EBs have also been standardized and applied as a promising alternative for cell transplantation [119,120]. Additionally, NSCs can be generated in monolayer cultures using serum-free specific inhibitors and growth factors that contribute to neuronal specification [121,122]. Overall, when comparing both techniques there are no major differences on the typical expression markers of NSCs and morphology. Nevertheless, there are some drawbacks associated with both assays. The EBs are 3-dimensional (3-D) spheroids structures capable of spontaneous differentiation mimicking better the embryonic development by promoting a heterogeneous differentiation, which is also a synonym of low reproducibility of the technique [123,124]. On the other hand, monolayer cultures need continuous passaging to maintain self-renewal and cell potency. 2-D attachment also interferes with the shape and geometry of colonies, which might be deleterious for internal cytoskeleton shape [125,126]. 

***Transdifferentiation of NSCs from somatic cells.*** Transdifferentiation is the differentiation of cells to a certain cell-type that does not follow the “normal” programmed differentiation mechanism [127]. This term was first applied by Selman and Kafatos (1974) and it is characterized by a direct reprogramming or conversion of one mature somatic cell type into another cell type without undergoing an intermediate pluripotent state [128]. Lineage reprogramming is mainly induced by the expression of endogenous lineage-specific transcription factors (TFs) [129] and by specific chemical compounds [130]. Yao et al. (2015) produced transdifferentiated induced NSCs (iNSCs) after conditionally overexpressing specific TFs (Oct4, Sox2, Klf4, c-Myc) from mouse embryonic fibroblasts. The transdifferentiated cells differentiated into mature astrocytes, neurons, and oligodendrocytes in vitro, and after being transplanted into a stroke rat model, they were able to reduce the lesion size and promote the recovery of motor and sensory function [131]. The TF-induced transdifferentiation has inherent issues associated with the use of exogenous viruses, so, chemical compounds [132] and growth factor-induced transdifferentiation [133] have been developed ensuring advantages in terms of safety. Transdifferentiation has become a powerful tool to study how cells might be manipulated for specific therapeutic purposes [134]. Nevertheless, this field is very recent and further investigation is still needed before the development of personalized regenerative therapies.

### 2.3. Advances on NSC-based Therapy for SCI

In 1999, McDonald et al. were the first to demonstrate the potentialities of NPCs transplantation in a SCI context. After deriving neural progenitors from mouse ESCs (mESCs) and transplanting them into a rat spinal cord 9 days post-injury they observed that grafts had the capacity to survive and differentiate into neurons, oligodendrocytes, and astrocytes, and to migrate along the rostro-caudal axis from the lesion epicenter. More importantly, the transplanted experimental group showed hind limb weight support and partial walk coordination [135]. From then on, numerous studies have been deciphering the mechanisms behind NSCs effects on SCI. However, there are still some critical questions that remain to be answered and should be addressed, such as, the optimal time-window of efficacy, the number of transplanted cells, the cellular source, safety, and administration routes. 

After grafting mouse fetal striatal NS/PCs 12 wpi into a contusion T10 mice model, Kumamaru et al. (2013) observed that cells were capable of releasing multiple regenerative molecules and to differentiate into neurons/oligodendrocytes contributing for a neurogenic spinal cord environment, however, no locomotor improvements were observed [136]. Efforts were also done by Cheng et al. (2017) to explore the appropriate timing for NSCs administration. After injecting hNSCs in a contusion T10 SCI model 1 or 4 wpi, both groups showed significant functional improvement at the motor level, but the effect was more prominent in the acute 1 wpi group [137]. Using NOD/SCID mice, after a T9 injury model, hCNS-derived NSCs were immediately injected after the inflicted injury and no locomotor recovery was observed [138], on the other hand, when Salazar et al. (2010) only applied the cells 4 wpi mice displayed locomotor improvements [139]. Despite the disparities, cells differentiate into all the three CNS lineages, survived, and migrated within the injured spinal cord suggesting that hCNS-derived NSCs transplantation can be effective depending on the time-window of intervention. 

Injecting the cells in an earlier phase might be more beneficial to restore the damaged neural circuits before the formation of the glial scar barrier. Nevertheless, the current number of chronic patients is highly considerable which should also stimulate the field to get novel strategies and boost cell transplantation effects in a more chronic phase. Yet, more preclinical studies are needed to better understand the best time window of NSCs transplantation. As mention above, SCI is a two-phase injury process with multiphasic cellular and molecular responses that varies along time which makes it difficult to find the best time-window of treatment [133]. To tackle such oscillations with cell transplantation between acute and chronic SCI, different protocols must be designed. For instance, in the acute phase NSCs may need to be transplanted together with some neuroprotective drug because several biological events are happening in this phase that may hamper cell survival. On the other hand, NSCs transplanted in the chronic phase may need the help of drugs that degrade the glial scar in order to better integrate in the host tissue.

The cellular source to obtain NSCs is also an important variable to be considered. First, animal studies must be carried out to better comprehend and explore the cellular viability and to decipher some potential targets of NSCs after transplantation. Further, preclinical studies are mandatory before conducting a clinical trial mainly to assess safety. Fetal brain and spinal cord are the two main sources used to generate viable NSCs. Using mouse fetal cortex, Cheng et al. (2016) injected NSCs in an acute contusion SCI mice 7 dpi and observed a reduction in M1 macrophages activation, neutrophils and iNOS^+^/Mac-2^+^ cells at the epicenter of the injured area. Although the underlying factors remain unknown, this beneficial anti-inflammatory profile was translated into a significantly enhanced neurological function in mice [140]. Additionally, different studies have also reported some locomotor improvements after chronic transplantation of NSCs using different cellular backgrounds from the mouse striatum [141], to shrew fetal hippocampus [142], to the rat fetal brain [143]. 

Concerning human fetal spinal cord NSCs, positive results have also been reported regarding axonal growth at the injured site. For instance, Kadoya et al., showed that after transplantation there is an extensive regeneration of the corticospinal axons, improving synaptic connectivity and forelimb functioning [144]. Meanwhile, Robinson et al. (2017) added to E14 spinal cord-derived NPCs a 4-factor cocktail (BDNF, bFGF, VEGF, MDL) which promoted significant maintenance of grafts survival and neural differentiation, fulfilling the lesion site [145]. 

The capacity of pluripotent mESCs to differentiate into the neural lineage has also been explored to reestablish the damaged circuits after a SCI. In a contusive SCI model, NSCs derived from a mESC line J1 were intrathecally transplanted 3 wpi showing the ability to differentiate into spinal GABAergic neurons and to attenuate chronic neuropathic pain [146]. In a moderate compression injury of the spinal cord, Salewski et al. (2015) performed acute transplantation of mESC-derived NSCs and the treated group showed significant functional improvements 8 wpi. During the differentiation protocol used, the group described an intermediate step of LIF-dependent neurospheres formation, named as primitive NSCs (pNSCs). Interestingly, they transplanted pNSCs to assess tumorigenic potential which resulted in teratoma formation, raising the warning for tumor formation potential of mESC-derived NSCs as a real safety concern that must be explored during differentiation protocols [53].

Furthermore, preclinical studies have been conducted, approaching different cell sources, to better comprehend better the cellular response after transplantation namely immune rejection, tumor risk formation, and grafts survival. Using a human primary source of neural precursors from the fetal spinal cord (SPC-01) it was showed strong immunomodulatory properties through astroglial p65 NF-κB inhibition which significantly impacted the reduction of the glial scar and the cavity size [147]. Moreover, the well-defined human H9 ESC-derived NSC line has also been commonly addressed as a cellular source with high reproducibility. These cells are characterized by the expression of NSC-specific markers, Nestin and SOX2, and the ability to differentiate into neurons, oligodendrocytes, and astrocytes [148]. After its transplantation to immunodeficient rats, the grafts showed signs of a continuous maturation over a follow-up of 1.5 years. After 3 months, mature neuronal markers were the first to be expressed, 6 months later mature astrocytes showed up, and only 1 year after transplantation mature oligodendrocytes were detected. NSC-derived astrocytes migrated longer-distances in the lesion site when compared with other cell-types and synergistically engrafted with host astrocytes resulting in modest improvements in forelimb motor function without any critical adverse outcomes [149,150].

On the other hand, human pluripotent cells have been yielding in the generation and maintenance of NSCs. Kumamaru and coworkers demonstrated that under specific cues human ESCs (hESCs) can be derived into NSCs committed to the spinal cord improving the potential of neural cells to restore the damaged circuities. After transplantation, cellular grafts revealed to be enriched in excitatory neurons, promoting a robust corticospinal regeneration, engraftment within the host increased synaptic formation, which has culminated in hind-limb improvements [151].

The discovery of iPSCs was one of the most remarkable innovations in regenerative medicine and biological research. The concept of convert adult somatic cells, such as blood cells or skin fibroblasts, into NSCs through iPSCs incited numerous studies in the field. Promising results have been observed after iPSC-derived NSC transplantation into SCI animal models, promoting cell survival, tissue preservation, and neuronal differentiation of cells. Moreover, functional recovery was also observed through the remyelination of axons and upregulation of supportive neurotrophic factors in the spinal cord [53,152,153].

Summing up, animal studies have been very useful to evaluate and decipher novel mechanistic insights after cell transplantation, yet preclinical studies are essential to target critical questions as immune rejection and tumor formation. To circumvent ethical issues and immunosuppression, iPSCs are now the most attractive human cell source, but further studies are required to ensure efficacy, feasibility and safety.

After getting a valid cellular source other questions need to be addressed, namely, the following question raised is, how many cells are needed for a positive outcome? The optimal number of transplanted cells is still very debatable and few studies have been in order to elucidate the field. Yousefifard et al. (2016) have recently performed recently a systemic and meta-analysis to tackle some of these questions regarding NS/PCs transplantation. They reported that the median number of the cells per kilograms of animal’s body weight was 4.3106 (interquartile range = 1.1106–2107), but the higher the number of transplanted cells the better was the functional recovery [154,155]. This might be related with a higher chance of cellular survival, and in addition, it was also observed that the number of transplanted cells improves hyperalgesia in the animals [155]. Therefore, it would be important to further study the optimal range of transplanted cells in order to facilitated the translation to humans.

The tumorigenicity risk associated with NSCs transplantation is also a real concern that must be addressed to ensure patients’ safety. While some studies do not report any tumor formation [139,156] others documented tumorigenic risk mainly associated with pluripotent-derived NSCs [157,158]. Moreover, as the field advances, evidence has been accumulating on iPSC-derived NSCs tumorigenicity that must be addressed to ensure high clinical quality in the application of these strategies. There are two main forms of tumorigenicity, teratomas and true tumors formation [159], yet the mechanisms triggered behind each of them are not fully understood. Some studies reported the use of tumor-inducing reprogramming factors and some residual undifferentiated cells as the main causes that induce epigenetic alterations in iPSC-derived NSCs [93,94]. 

Teratomas development is mainly associated with an existent “epigenetic memory” and the lack of purification in the cellular sample used for transplantation [93]. Some strategies that can be undertaken are: increasing the number of passages to dissolve the “epigenetic memory”; develop purification systems with higher yield; reprogram iPSCs to drive away from the teratoma-inducing lineage; or even transplant cells in a more differentiated state. 

To avoid tumor formation during iPSCs reprogramming and differentiation there are some critical steps that must be carefully performed to avoid epigenetic and genomic instability in colonies [94]. The choice of the reprogramming method is a key-step to avoid genome disturbance by choosing integration-free systems over integrative vectors [101,102]. Further, the selection of the reprogramming factors is also critical, since c-Myc from Yamanaka’s factors is by itself sufficient to induce tumorigenesis [90,100]. However, it was already showed that c-Myc is not indispensable for iPSCs generation, avoiding its application [99,100,160]. Recently, based on these withdraws, Kojima et al. (2018) introduced a specific gene into a tumorigenic human iPSC-NSCs line to successfully ablate immature proliferating cells, and after transplantation, animals did not develop any tumor, moreover, this cells promoted motor recovery [156]. 

The route of administration is also an important topic that must be considered when addressing cell transplantation. Three main injection routes have been tested to be applied in SCI context: intraspinal, intrathecal, and intravenous. Amemori et al. (2015) studied different administration routes in an acute SCI model. NSCs were either injected intraspinally into the lesion center or intrathecally into the subarachnoid space of rats with compression lesion. Both treatments facilitated functional locomotor recovery, yet the intraspinal implantation had a higher positive effect in gray and white matter sparing and axonal sprouting, and reduced astrogliosis when compared to the intrathecal injection [152]. Nevertheless, when Cheng et al. (2012) injected hNSCs both locally at the injury site or distally no significant differences were observed in functional behavior [161]. 

Intraspinal injections are the most commonly used route by researchers, however, it is important to have in consideration some positive results published regarding intravenous administration [162,163]. Nishimura’s et al. (2013) reported that after hNSC intravenous administration animals showed behavioral improvements, electrophysiological recovery, suppression of glial scar formation, and preservation of nerve fibers. These results suggest that cells are able to survive, proliferate, and migrate into the lesion site [163]. Moreover, Osaka et al. (2010) also support intravenous administration of cells as a minimally invasive approach with high therapeutic potential [164]. Once again, more investigation should be performed considering the ideal route of administration. Minimal invasive administration procedures avoid surgical complications to patients, however, we may also lose some therapeutic efficacy. All these factors should be well studied in the preclinical set up.

On Table 2 it is represented selected preclinical studies employing NSCs for SCI repair. Overall, from a morphological point of view, NSCs are able to differentiate into neurons, astrocytes, and oligodendrocytes promoting axonal regrowth, remyelination and regeneration of the CST [145,150,165]. In addition, NSCs grafts showed consistently the capacity of filling the lesion cavity, reducing the glial scar and a high chance of survive after transplantation [144,166]. Physiologically, Kumamaru et al. (2018) showed that differentiated cells from NSCs form new synapses within the host spinal cord below the lesion. After assessing cell grafts, they also observed that cells present mainly an excitatory neuronal fate which promotes the host-to-graft connectivity [151]. Moreover, NSCs transplantation has consistently demonstrated to promote functional motor recovery in SCI animal models. For instance, Kadoya et al. (2016) demonstrated that transplanted rats have an improved motor function, by performing the staircase task, which is a supraspinal dependent test and requires a skilled forelimb control [144]. Lu et al. (2017) also reported that even 18 months after NSCs transplantation, cells differentiate and promote functional recovery [150]. Finally, and particularly important, the vast majority of the studies transplanted human-derived NSCs into animal models, which imposes the use of immunosuppressant drugs in combination with the cells. From the immunological point of view, several studies showed that when exogenous NSCs are combined with immunosuppressant drugs they can successfully integrate in the rat/mouse spinal cord without immune rejection [27,166,167]. However, data from clinical trials have shown that when the immunosuppressant therapy is over, patients experience complications related with immune rejection of the new tissue (please see Section 2.4) [168]. For this reason, the development of iPSCs-based therapies is very important to minimize the probabilities of immune rejection by the patient. Therefore, differentiation of NSCs from iPSCs allows to acquire a patient-specific source of suitable cells that can differentiate into neuronal lineages with the aim to replace and assist the damaged neurons. 

Due to the complexity of the SCI pathophysiology, different approaches have been developed combining cell-based transplantation with other target-therapies. For instance, after combining chondroitinase ABC (ChABC) treatment with iPSC-derived NSCs mice showed improvements in the forelimb grip strength and forelimb/hindlimb locomotion. The cells were injected after injury, but the ChABC treatment was only administrated 7 wpi. Interestingly, the grafts showed improved survival and differentiation potentially promoting functional synaptic connectivity [27]. With a different strategy, Okubo et al. (2018) injected gamma-secretase inhibitor (GSI)-treated hiPSC-NS/PC into a chronic SCI model. After injection mice showed a significant increase in axonal growth, remyelination, inhibitory synapse formation within the host neural circuitry, and reticulospinal tract fiber formation. In fact, previous work from the group, demonstrated that GSI treatment inhibits Notch signaling promoting neuronal differentiation [169]. Finally, combining these favorable factors leads to motor improvements in lesioned animals [165]. 

Moreover, biomaterials have also been currently applied as artificial extracellular matrix with the aim to increase the survival of transplanted stem cells. Using a laminin-coated hydrogel with dual porosity, iPSC-NPs were seeded and further transplanted into a rat model of chronic SCI. The cells survived over time and promoted the growth of host axons, astrocytes, and blood vessels; however, no locomotor recovery was observed [166]. 

Overall, a vast number of approaches have been employed to achieve the best outcome and a significant regeneration of the damaged neurons after SCI, however, some challenges remain to be overcome. Cell-based therapy is a promising strategy since it is capable to target different events, allowing a neuroprotective environment and regenerative support in the injured spinal cord. Therefore, regeneration of the adult spinal cord cannot be thought as a one-step process, but rather as a multiple cascade-process overtime.

### 2.4. SCI Clinical Trials Based on NSCs

Translational medicine has been one of the biggest challenges in science and medicine. Interestingly, the European Society for Translational Medicine (EUSTM) defined it as *an interdisciplinary branch of the biomedical field supported by three main pillars: benchside, bedside, and community* [173]. Promising studies have been developed concerning NSCs transplantation to different SCI models, inciting now an important and necessary transition from the *benchside*-to-*bedside*. Although the encouraging results reported in preclinical studies, translation into the clinical set is still very challenging [174], being the main limitations: anatomical differences between experimental animal models and human SCI [175]; inconsistency observed in the therapeutic efficacy; variability in NSCs generation; number of transplanted cells; logistics to obtain donor cells that can be reliable and safely stored for clinical use; ethical concerns; sample size and subject selection criteria; absence of standardization in the post-assessment tests; and extensive costs of running clinical trials. 

To date, a low number of clinical trials have been conducted for SCI patients using NSCs, nevertheless, some have already been concluded and others are still ongoing (Table 3). 

The first clinical trial using NSCs was approved in 2005 by the Yonsei University Health System, Severance Hospital, Republic of Korea (Clinical Research Information Service (CRIS), Registration Number: KCT0000879). The phase I/ II clinical trial was based on the transplantation of NSC-derived from brain fetal tissue. Human NSCs neurospheres between P10–P20 were carefully selected and prepared for transplantation into 19 patients which were subdivided into four groups according to the time window between the injury onset and transplantation: acute (<1 week), early subacute (1–8 weeks), late subacute (9 weeks–6 months), and chronic (>6 months). A control group was also addressed to the clinical trial of 15 patients with traumatic cervical SCI. After 1 year, the study concluded that hNSCs transplantation is safe and well-tolerable by patients since no adverse evidence was observed. The patients did not exhibit evidence of cord damage, syrinx or tumor formation, neither neurological deterioration nor exacerbating neuropathic pain and spasticity. Regarding neurological outcomes, based on the American Spinal Injury Association Impairment Scale (AIS) grade, 5 out of 19 transplanted patients showed beneficial alterations, including recovery at the motor level and increased motor scores, whereas only 1 patient in the control group showed improvement [176]. 

Neuralstem Inc. (Germantown, MD, USA) began a phase I safety trial (clinicaltrials.gov identifier: NCT01772810) at the University of California San Diego to intramedullary injected an FDA-approved NSI-566RSC cell line into thoracic (T2-T12) SCI subjects. NSI-566RSC line is derived from the cervical and upper thoracic regions of the spinal cord from an 8 gestational weeks human fetus. The first report was published by Curtis et al. (2018) showing that NSI-566 grafts are safe and have no detectable side effects after 18–27 months of cell transplantation [178]. However, the small sample size (n = 4) is a major drawback in the assessment of this one-open-arm study. Nevertheless, future studies are needed and justified due to the positive results regarding the safety and tolerability of NSI-566RSC cells in subjects. 

As mentioned before, Asterias Biotherapeutics Inc. (Fremont, CA, USA) has also begun a safety trial in the implementation of human ESC-derived oligodendrocyte progenitors (GRNOPC1 cells) [78] in patients with neurologically complete subacute SCI (clinicaltrials.gov identifier no. NCT01217008). Although some financial problems delay the initial study, a second clinical trial was initiated as a phase I/IIa dose-escalation study using the same embryonic-derived OPCs (NCT02302157). Preliminary results suggest improvements in motor function, but further data still needed [80].

In 2014, a different clinical trial was also initiated to evaluate the safety and the potential of directly reprogrammed autologous NSCs (drNSCs) in subacute and chronic SCI subjects (Federal Research Clinical Center of Federal Medical & Biological Agency, Russia, NCT02326662). Directly reprogrammed NSCs were generated from patients’ bone marrow cells (BMCs) using a patented reprogramming xeno-free cocktail. The grafts were directly injected into the white and grey matter of the spinal cord adjacent to the lesion after the implantation of a regeneration matrix (RMx). Although the sample size presented by the study is extremely limited (n = 5), after 6–9 months of follow-up there was no complications or side effects associated with the intervention, and all patients showed improvements in the neurologic state. 

Many different reports have already described the benefits of research cell lines transplantation into rodent models of thoracic contusion SCI [185,186,187,188]. Concomitantly to these results, Stemcells, Inc. led a phase II clinical trial in thoracic SCI patients using the HuCNS-SCs cell line (NCT02163876). The main aim was to escalate the dose safety and efficacy, 4 months after injury, of intramedullary injections rostral and caudally to C5-C7 injury. An optimistic press release of interim 6-month data (November 18, 2015) reported improvements in motor strength in 4 out of 5 subjects. Using the same HuCNS-SCs, Anderson et al. (2017) inject them in an in vivo preclinical study to validate locomotor and sensory recovery, cell engraftment, migration, and neural lineage fate of cells [48]. Yet, no evidence of HuCNS-SCs efficacy was observed raising awareness for the necessity of validation of cell lines and/or in cell manufacture/processing, to diminish possible variations and ensure consistency along preclinical studies to clinical trials. Nevertheless, after one-year post-transplantation, no evidence of additional spinal cord damage, new lesions, or syrinx formation was observed in patients. An efficacy threshold was previously set by the sponsor to further support the study and by citing a lack of significant improvements and trend for improvements over time, StemCells Inc. actually terminated the study [189].

In 2011, a new phase I/II clinical trial (NCT01321333) was sponsored by Stem Cells Inc. and managed at the University Hospital Balgrist (Zurich, Switzerland). Based on previous preclinical studies, the main focus was to assess the safety and preliminary efficacy of human CNS stem cells (HuCNS-SC) transplantation. The trial included 12 thoracic (T2-T11) SCI patients who sustained an injury within 3–12 months prior to cell transplantation. At the injection time, patients received approximately a total of 20 million cells directly into the spinal cord, 2 injections rostral and 2 caudally to the injury site. After 12-months o, HuCNS-SC showed to be safe and feasible concerning the surgery and the cellular transplant. More interestingly, 7 out of the 12 patients experienced sensory improvements after neurological stability [178]. 

Following these positive results, a second clinical trial was approved (NCT01725880) to perform a long-term follow-up of the transplanted HuCNS-SC subjects. However, some obstacles appeared that should be carefully considered. After 9 months of immunosuppression, the agents were removed and the sensory gains were lost. It seems that the immune system was rejecting the transplanted cells which culminate with the ceasing of the long-term follow up. Although the result observed in SCI patients was not expected, this warned the field for a real problem in transplant non-patient cells that might be challenging the host immune system. Consequently, the cell source used for clinical cell transplantation must be rethought being NSCs-derived from iPSCs one of the most attractive alternatives.

Okano’s team in Japan is proposing the launch of the human clinical study using allogenic iPSC-derived NSCs for subacute SCI patients. The costs associated with quality testing, safety concerns and time-consuming for autologous transplants have led the team to an increased interest in an allogeneic alternative. Therefore, the Center for iPS Cell Research and Application (CiRA) at Kyoto University will donate “iPSCs of human leucocyte antigen (HLA) super-donors” for cell transplant intervention, which are clinical-grade allogenic immunologically match clones and no adverse outcomes are expected. Thus, the conduction of this clinical trial intends firstly to address safety and tumorigenesis issues associated with the transplantation of these clones into humans as a future viable therapeutic intervention [190,191].

Overall, clinical trials have been showing some modest improvements to the SCI patients. More important, these trials have not shown major safety problems, however the clinical trial perform by University Hospital Balgrist shown that after stopping immunosuppression complications may arise. For these reasons, autologous iPSC-derived NSCs are the most promising cell source to repair the injured spinal cord. This section was only based on the NSCs-based, however, several different therapeutic agents were or are being testing in clinical settings with the aim to promote SCI repair. For more information about clinical trials in a SCI context please read the following reviews [192,193,194,195].

## 3. Future Perspectives

NSCs are a promising therapeutic approach for SCI repair. NSCs can differentiate and replace the lost neural tissue as well as secrete neurotrophic factors that can protect or regenerate the tissue. Nevertheless, further studies are necessary to confirm neurological and functional benefits, safety, adjusting doses and administrations periods, and to select the most promising cellular sources to obtain NSCs. Despite all the efforts and progress, the match between preclinical models and human SCI is still poorly established but several ongoing clinical can provide an important avenue for future cell transplantation research. It is also important to highlight that cellular transplantation alone may not be a sufficient approach to completely treat the injured spinal cord, therefore, combinatory therapies may be necessary for the treatment of this devastating injury. 

## Figures and Tables

**Table 1:** Overview of the advantages and limitations of induced pluripotent stem cells generation and establishment from somatic cells.

Advantages	Limitations
No ethical concerns	Risk of tumor formation
Evidence on a pluripotent profile (ES-like cells)	Epigenetic and genetic alterations
Highly flexible technique	Oncogenes reactivation
Derivation from any somatic cell	Expensive and time-consuming
Patient-specific source (no immune rejection)	

**Table 2:** Preclinical studies using neural stem cells transplantation to target spinal cord injury repair.

SCI	Animal Model	Injury	Transplanted Cells	Time	Additional Treatments	Outcomes	REF
Transection	Fischer 344 Rat C57BL/6 Mice	T3 C4	Rat E14 SC-derived NPCs Mouse E12 SC-derived cells	2 w		Cell grafts survival Full-fill of the cavitation site Axonal CST regeneration and functional synaptic formation Improved forelimb function	[144]
Contusion	C57BL/6 Mice	T9/10	Mouse Fetal Brain NSCs	1 w		Migration from the injection site toward the injury Locomotor improvement Reduction in neutrophils and iNOS^+^/Mac-2^+^ cells Downregulation of TNF-α, IL-1β, IL-6 and IL-12	[170]
Hemisection	Fischer 344 Rat	C5	Rat E14 SC-derived NPCs	2 w	4-factor cocktail	Consistent graft survival Neuronal differentiation Reduction of the lesion site	[145]
Compression	Wistar Rat	T10	Human Fetal Spinal Cord SPC-01 cell line	1 w		Downregulation of TNF-α Inhibition of p65 NF-κB Reduction of glial scar and cavity size Gray matter preservation	[147]
Compression	C57BL/6 Mice	T6	ES-dNSC	1 w		Enhancement of spared neural tissue Differentiation into oligodendrocytes Motor improvement	[171]
Hemisection	Nude Rat	C5	H9 ESC-derived NSCs	2 w		Graft size stable over time Differentiation into mature neurons and glia Long axonal regrowth Glial migration to host white matter	[150]
Contusion	NOD-scid Mice	T9	hCNS-derived NSCs	0		Astroglial differentiation of donor cells in the lesion site No locomotor recovery	[138]
Transection	Nude Rat	C4	hPSC-derived Spinal Cord NSCs	2 w		NSCs committed to a spinal cord phenotype Differentiation into excitatory neurons Regeneration of the CST Host-to-graft synaptic connectivity	[151]
Compression	WT Mouse C3Fe.SWV-Mbpshi/J Mice	T6	iPS-derived NSCs	1 w		Integration within the lesion site Differentiation to oligodendrocytes *wt*-iPS-dNSCs promote remyelination and axonal function Motor Improvements	[53]
Compression	Wistar Rat	T8	iPS-derived NPs	1 w		Intraspinal implantation promote: > gray and white matter sparing > axonal sprouting > astrogliosis reduction Moderate functional recovery	[152]
Compression	Wistar Rat	T8/T9	hiPSC-derived NPs	1 w		Cell survival and tissue preservation Differentiation into the three germ layers Motor improvement Increased expression of NFs Neuronal regeneration	[153]
Contusion	C57BL/6 Mice	T10	iPSC-derived NPCs	1 w		Neuronal lineage differentiation No tumor formation No locomotor recovery	[167]
Contusion	Long-Evans hooded Rat	T10	Human Fetal Brain NSCs	4 w		Trophic effect in the CSF Motor improvement	[140]
Contusion	C57BL/6 Mice	T9	Mouse Striatal NS/PCs	7–10 d	Treadmill Training	Differentiation into neurons, oligodendrocytes, and astrocytes Electrophysiologic recovery Locomotor improvements	[141]
Contusion	Rat	T10	Rat Spinal Cord NSCs	13 w	Ch combined with NFs	60% of survival < 40% of the lesion site covered Improvement in bladder function	[172]
Hemisection	Tree Shrew	T10	Shrew Fetal NSCs	9 d		Self-renewal potential Differentiation into neurons and astrocytes Production of NFs (CNTF, TGF-β1, GDNF, NGF, BDNF and IGF)	[142]
Contusion	Wistar Rat	C6/C7	Rat Fetal Brain NSCs	10 d		Long-term survival Differentiation along the oligodendroglial lineage Reduction in M1 macrophages Lower density of iNOS Functional recovery Reduction in apoptosis	[143]
Contusion	Sprague–Dawley Rat	T12	mESC-derived NPCs	3 w		In vitro differentiation into a spinal GABAergic phenotype Attenuation of chronic neuropathic pain	[146]
Hemisection	Nude Rat	C5	Human H9 ESC-derived NSCs	2 w		No cellular migration Improvement in skilled forelimb motor function	[149]
Contusion	C57BL/6 Mice	C6/C7	iPS-derived NSCs	8 w	Intrathecal ChABC	Cell survival Remyelination and synaptic formation Behavioral recovery of the forelimb grip strength and locomotion	[27]
Contusion	NOD-SCID Mice	T10	hiPSC-derived NS/PCs	6 w	GSI	Axonal regrowth and remyelination Reticulo-spinal tract fiber formation Motor functional recovery	[165]
Compression	Wistar Rat	T8/T8	hiPSC-derived NS/PCs	5 w	Laminin-coated pHEMA-MOETACl hydrogel	Survival and integration within the lesion spinal cord Reduction in cavity depth and axonal growth Increased number of astrocytes, blood vessels, and TH^+^ fibers No locomotor recovery	[166]

Abbreviations: w weeks; d days; wt wild-type; ChABC chondroitinase ABC; NTFs neurotrophic factors; GSI gamma-secretase inhibitor; CST corticospinal tract; BDNF brain-derived neurotrophic Factor; bFGF basic-fibroblastic growth Factor; VEGF vascular endothelial growth factor; NSC neural stem cell; NPC neural progenitor cell; iPSC-derived NSC induced pluripotent stem cell-derived NSCs; mESC-derived NPCs mouse embryonic stem cell–derived NPCs; hiPSC-derived NS/PCs human induced pluripotent stem cell-derived NS/PCs; ES-dNSC embryonic stem-definitive NSCs; hCNS-derived NSCs human central nervous system-derived NSCs.

**Table 3:** Spinal Cord Injury Clinical Trials using NSC-based therapies.

Start Year	Sponsor	Country	NTC/I.D.	Clinical Phase	SCI Cohort	Cell-Type	Cell Source	Safety	Improvements	Others	REF
2005	Yonsei University Health System, Severance Hospital	KR	KCT0000879	Phase I/II	Cervical	hNSPCs	Fetal brain	Safe and well-tolerable	Partial sensorimotor function	No cord damage, syrinx or tumor formation No neurological deterioration, and exacerbating neuropathic pain or spasticity Incomplete sensory recovery	[176]
2011	StemCells, Inc.	CAN CH	NCT01321333	Phase I/II	T2-T11	HuCNS-SCs	Fetal brain	Safe and well-tolerable	Segmental sensory	Decline in sensory gains lost after withdrawal of the immunosuppressive	[168]
2012	StemCells, Inc.	CH	NCT01725880	Phase I/II	T2-T11	HuCNS-SCs	Fetal brain	Study terminated based on a business decision			[177]
2013	Neuralstem Inc.	US	NCT01772810	Phase I	T2-T12	NSI-566 cell line	Fetal spinal cord (cervical and upper thoracic regions)	Safe and no side effects 18–27 months after cell delivery		Low sample size (n = 4) Still Recruiting	[178]
2014	StemCells, Inc.	US CAN	NCT02163876	Phase I/II	C5-C7	HuCNS-SCs	Fetal brain	Slight motor strength but the study was terminated based on a business decision			[179]
2017	University of Zurich	CH	NCT03069404	Phase I/II	T2-T11	HuCNS-SCs	Fetal brain	No data			[180]
2014	Federal Research Clinical Center of Federal Medical & Biological Agency	RU	NCT02326662	Phase I/II	Neck, thoracic or lumbar	drNSCs	BMCs	Safe with any complications	Neurologic state		[181]
2016	Chinese Academy of Sciences	CN	NCT02688049	Phase I/II	C5-T12	NSCs		No data Still recruiting			[182]
2010	Asterias Biotherapeutics	US	NCT01217008	Phase I	Neurologically Complete, Subacute	GRNOPC1	hESCs	The study was terminated based on financial issues			[183]
2015	Asterias Biotherapeutics	US	NCT02302157	Phase I/II	C4-C7	AST-OPC1	hESCs	Favorable safety profile	Some hand functions		[184]

**Abbreviations:** T thoracic; US United States; CAN Canada; CH Switzerland; CN China; RU Russian Federation; KR Republic of Korea; NSCs neural stem cells; hESCs human embryonic stem cells; BMCs bone marrow cells; HuCNS-SCs human central nervous system stem cells; NSI-566 cell line human spinal-cord-derived NSC; drNSC directly reprogrammed autologous NSCs; AST-OPC1 AST-oligodendrocyte progenitor cells; GRNOPC1 human embryonic stem cell-derived OPCs.
